# Instability treatment due to upper cervical tuberculous spondylitis

**DOI:** 10.1016/j.ijscr.2019.07.044

**Published:** 2019-07-23

**Authors:** Singkat Dohar Apul Lumban Tobing, Rendra Irawan, Mohammad Triadi Wijaya, Aji Antoro, Eko Setiawan, Rian Septian

**Affiliations:** a Spine Division, Department of Orthopaedic & Traumatology, Cipto Mangunkusumo National Central Hospital and Faculty of Medicine, Universitas Indonesia, Jalan Diponegoro No. 71, Central Jakarta, Jakarta 10430, Indonesia; b Department of Orthopaedic & Traumatology, Cipto Mangunkusumo National Central Hospital and Faculty of Medicine, Universitas Indonesia, Jalan Diponegoro No. 71, Central Jakarta, Jakarta 10430, Indonesia

**Keywords:** Upper cervical tuberculous spondylitis, Cervical infection pathophysiology, Outcome upper cervical tuberculous spondylitis

## Abstract

**Introduction:**

Upper Cervical Spondylitis is a rare case, approximately 0.3–1% of all Tuberculous Spondylitis. Nevertheless, it causes destruction and morbidity more than Tuberculous Spondylitis at other side. Instability at upper cervical make patient cannot perform loadbearing activity. Treatments consist of anterior debridement with fusion and posterior stabilization is one of treatment that present good outcome. This study has been reported in line with the SCARE criteria (Fang et al., 1983) [1].

**Material and method:**

We collected the data A 22 years old young man with chief complaint of severe pain on his neck since 1 year before admission. And this work has been reported in line with the SCARE criteria. He was diagnosed Tuberculous Spondylitis at C1–C2 and C6–C7 and got debridement, decompression and posterior stabilization and fusion also with occipital plate, lateral mass and long rods.

**Result:**

Performing debridement, decompression, posterior stabilization and fusion using occipital plate, lateral mass and rods as a treatment for Upper Cervical Tuberculous Spondylitis give promising outcome. For increasing functional outcome, patient got sensoric and motoric exercise by physiotherapy

**Conclusion:**

Upper cervical treatment with debridement, decompression, posterior stabilization and fusion using occipital plate, lateral mass and rods give optimal outcome.

## Highlights

• Spine is the most common extrapulmonary tuberculosis manifestation, with predilection in thoracal and lumbal.
• Cervical manifestation, although very rare, could elicit instability and neurological deficit.
• Single step surgery shows early strong stabilization, prevention of complication and reduction of using halo post-surgery.

## 1 Introduction

In underdeveloped country, tuberculosis still common [1]. The most common extra pulmonary manifestation is in spine, especially thoracal and lumbal [2]. Its extra pulmonary manifestation in upper cervical still rare, but can give serious condition. There are 2–3% instability and neurological deficit due to tuberculosis lesion. Only 0.3–1% atlantoaxial tuberculosis (AATB) among all of Tuberculous Spondylitis, and this case was reputed as very rare case [3].

There are 3 ways tuberculosis can affect spinal cord at medullary cervical junction: atlanto-axial subluxation or translocation upside by dens, compression due to tuberculous abscess and tuberculosis direct invasion [1].

Single step surgery consists of anterior and posterior in one time showing achieve early strong stabilization, prevent complication due to graft and reduce using of halo post-surgery. Fusion time can be decreased by using this method also [4,5].

## 2 Case illustration

We collected the data A 22 years old young man with chief complaint of severe pain on his neck since 1 year before admission. And this work has been reported in line with the SCARE criteria [6]. He also complains about numbness at all of his body. After couple of days, he felt lack of power and tingling at his upper and lower extremity without abnormally of the reflexes. He got traditional treatment by message on his neck. After 3 months of traditional treatment, he felt that his complain was getting worse. He couldn’t move his neck, especially on the left side. It was local pain, and his extremity function is good. There was no improvement from conservative management.

From laboratory results, there was an increase of ESR (33 mm), leukocytosis (15.86 × 10^3^/μL), and C *Reactive Protein* (23.6 mg/L). There was negative result in Ziehl Nielsen staining, maybe because of too much saliva or inadequate sample. There was negative results in ICT TB also.

From radiology examination, On cervical x ray (Picture 1 ), we found pathological kyphosis in upper cervical. There was collapse of Cervical 1 and 2 and destruction of endplate, and swelling of surrounding soft tissue also. From MRI examination (Picture 1), we found a mass, well defined border, regular edge, hypo-intensed in T1, and hyper-intensed in T2 located in retropharyngeal at C1,2 level, and expand to posterior vertebra body (ring enhancement) after contrast. It gives pressure on spinal canal. Vertebral body, lamina, processus transversus and spinosus still intact. There was hyper-intensed in vertebra body C2, 7 and Th1.

**Picture 1:**
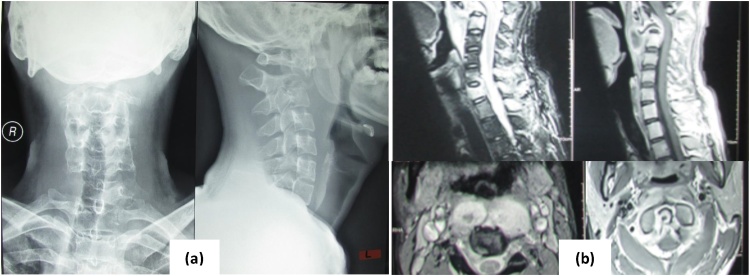
Radiology examination. (a) X-ray Cervical AP and Lateral. (b) MRI cervical sagittal and axial.

Patient was diagnosed with Multiple Tuberculous Spondylitis C1,2 and C6,7. We perform anterior debridement and posterior stabilization. Anterior stabilization by transoral incision, and continue by debridement with NaCL 0.9%. We found cold abscess from it (Picture 2 ). After perform anterior debridement, we change patient position to prone. We make incision from occipital to Th6. We make posterior stabilization at occipital, C2,4,5, Th1 and Th2 and we perform fusion with synthetic bone graft (Picture 2).

**Picture 2:**
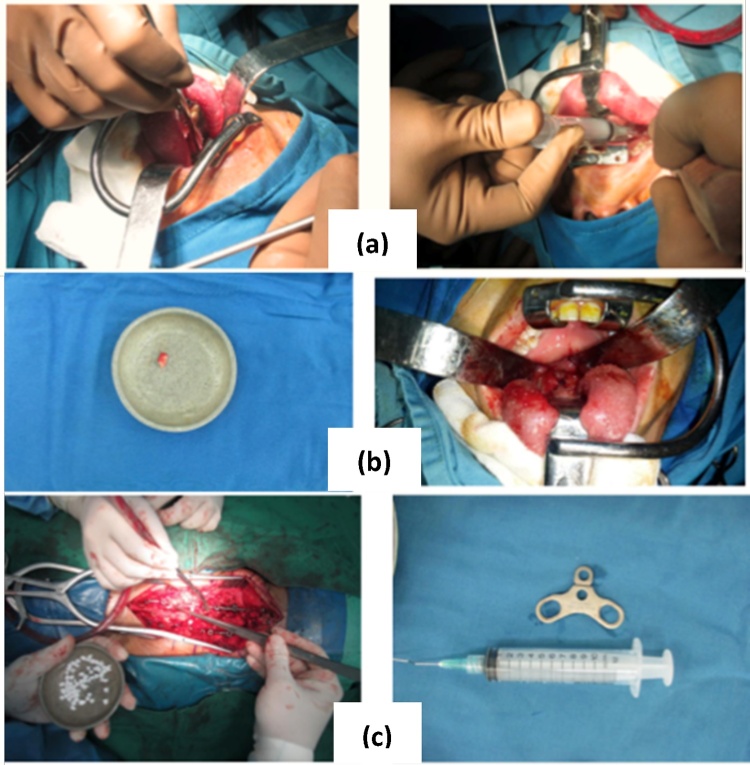
Intraoperative procedures. (a) Transoral incision and anterior debridement. (b) Cold abscess from anterior debridement. (c) Posterior stabilization at occipital level to C2,4,5 and Th 1,2 and fusion using synthetic bone graft.

From histopathology we found chronic cell and eosinophil. There are foam cells, and epitheloid cells, that commonly found in Tuberculous Spondylitis.

After performing surgery, patient was observed in ICU for 2 days, and discharge to common ward in 3^rd^ day. He can walk and do loadbearing activity in 5 days after surgery. He still uses collar brace to limit neck movement like rotation, flexion and extension. There was no neurological deficit after surgery. From post operative xray (Picture 3 ), the alignment was restored, with steady of posterior stabilization.

**Picture 3:**
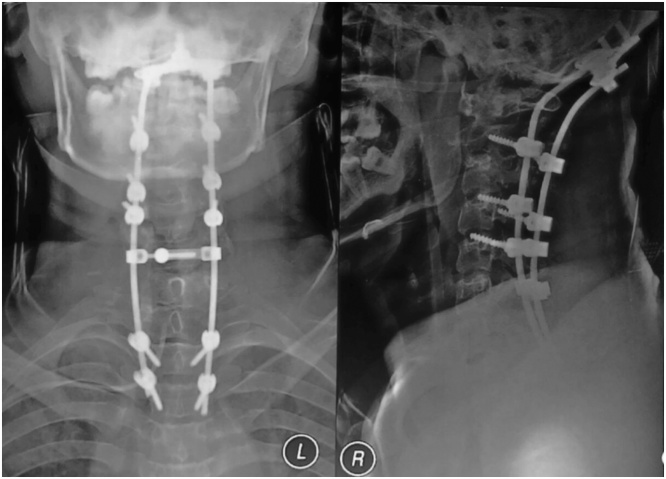
X-ray Cervical AP and Lateral after surgery.

## 3 Discussion

Tuberculosis of vertebrae has been found since 5000 years ago. A British Surgeon, Sir Perceival Pott is a first who report extrapulmonary tuberculosis that associated with paraplegia and spine kyphosis deformity [7]. Atlantoaxial tuberculosis*, AATB* is rarely diagnosed in tuberculosis of vertebrae, only 0.3–1% [8,9]. The most common cause of vertebrae infection is bloodstream associated infection. First, it starts around vertebra endplate, where vascularization decreases. After affected, all of endplate infected, and spread to disc and opposite vertebral endplate. If not treated, it will destruct bone and make instability and neurological deficit eventually [10]. In this patient, we found hemoptysis, bodyweight decrease, but previous lung tuberculosis history still unclear.

The most common site of vertebra being infected is paradiscal, but it can affect central of vertebral body, anterior (anterior type), pedicle (appendicular type), lamina, transversus processus, posterior intervertebral joint (articular type) [11]. Tuberculosis infection may cause destruction, necrotic in vertebrae or an abscess. AATB make extensive osteoligament destruction in the most mobile cervical [12]. Neurological compression, is a direct effect of instability or due to compression of mass [9,12,13]. Explanation about neurological deficit is common in cervical due to small size of cross sectional spinal column from spinal cord relatively [11]. If affecting cervical, the most common symptom is neck pain, and it can be detected 24 month earlier before the diagnosis is established. Bormann and Dunn reported neck pain and stiffness in all their serial case patients [3]. More than 50% patients reported muscle weakness [14]. They report 1 patient showing muscle weakness in left arm and alteration of sensation and reflex. Other patient report muscle weakness in all of extremity and increase of muscle tone and reflex [3].

Although standard laboratory examination like hematology, ESR, chemical blood, tuberculin test (Mantoux) was helpful, but it is not significant in making diagnosis [11]. In this patient, there was an increase of ESR, leukocytosis, CRP, and acid-resistant bacteria test was negative, maybe due to error in taking sample.

Radiology examination showed narrowing disc space in early phase, with loss of paradiscal border, it clearly showed in paradiscal type. Radiology examination also showed kyphosis and soft tissue shadow [16]. Lytic and destruction of vertebral body in early phase showed in CT, and showing paraspinal and granulation also [15,17]. MRI is useful to evaluate lesion position and its expansion that could compress soft tissue around it. Gupta et al reported that MRI could show abnormality in 63% of spinal tuberculosis patient with normal x-ray [18].

Optimal treatment for spinal tuberculosis is debatable and must be adjusted to patient condition [11]. Cervical tuberculosis with progressive neurological deficit with or without progressive deformity must be treated directly [19]. Gold standard treatment is decompression and anterior spine instrumentation for supporting collapse cervical [11,14,15]. If there was no deformity or neurological deficit, the treatment is only antituberculosis medicine, bed rest and suitable mobilization with orthosis [15]. Best treatment for cervical tuberculosis with paraplegia is prevention the progression of paraplegia [15]. It can be done in patient with progressive neck pain before neurological deficit [11]. Spontaneous fusion is rare in spondylitis, that can cause kyphosis and neurological deficit. Although medical treatment is a basic treatment, surgical intervention is common to increase treatment effect [11]. Surgery is indicated for diagnostic biopsy, abscess drainage, neural decompression, deformity correction and stabilization of affected spine [11]. Anterior debridement, using autograft bone is useful method to support bone healing locally and accelerate stability and prevent deformity. Instrumentation is not contraindicated if adequate debridement was performed and vascularization still normal [4]. This instrumentation reduces length of stay and prevents graft movement [11]. Hodgson, Stock et al reported 94% patient was totally recovered after anterior approach for decompression with autograft [21]. Posterior stabilization was adjustable as a support for anterior surgical approach. Anterior and posterior surgical combination in C3 tuberculosis showed stable and direct achievement, prevented graft complication and reduced halo use after surgery. Fusion rate was significantly increased if this surgical technique performed [4,5]. In this patient, we performed debridement and decompression from anterior by transoral incision, and continued by stabilization and fusion posteriorly. After post-surgery observation in ICU, patient could do loadbearing activity in 5^th^ day.

## 4 Conclusion

Our treatment for multiple cervical tuberculous spondylitis consisting of debridement and decompression from anterior by transoral incision followed by stabilization and fusion posteriorly resulted in optimal healing of the patient. The patient’s complaint of pain was relieved and his weakness was improved. We recommend this treatment regimen in treating spondylitis cervicalis.

## Funding

There is no sources of funding sponsor in this manuscript.

## Ethical approval

The authors have no ethical conflicts to disclose.

## Consent

The patient has given her informed consent for the case report and accompanying images to be published.

## Author contribution

1 Singkat Dohar Apul Lumban Tobing, MD. Contributed as making the conceptualization, data curation, study design, funding acquisition, supervision, and final approval of manuscript.
2 Rendra Irawan, MD. Contributed as making the study design, collecting, and analyzing the data, formal analysis, writing manuscript.
3 Mohammad Triadi, MD. Contributed as making the study design, collecting, and analyzing the data, formal analysis, writing manuscript.
4 Ajiantoro, MD. Contributed as making the study design, collecting, and analyzing the data, formal analysis, writing manuscript.
5 Eko Setiawan, MD. Contributed as making the study design, collecting, and analyzing the data, formal analysis, writing manuscript.
6 Rian Septian, MD. Contributed as making the study design, collecting, and analyzing the data, formal analysis, writing manuscript.

## Registration of research studies

Not available.

## Guarantor

Singkat Dohar Apul Lumban Tobing, MD.

## Provenance and peer review

Not commissioned, externally peer-reviewed.

The authors have no ethical conflicts to disclose.

## Declaration of Competing Interest

The authors have no ethical conflicts to disclose.
